# Subjective Quality Assessment of V-PCC-Compressed Dynamic Point Clouds Degraded by Packet Losses

**DOI:** 10.3390/s23125623

**Published:** 2023-06-15

**Authors:** Emil Dumic, Luis A. da Silva Cruz

**Affiliations:** 1 Department of Electrical Engineering, University North, 104. Brigade 3, 42000 Varaždin, Croatia; 2 Department of Electrical and Computer Engineering, University of Coimbra, 3030-290 Coimbra, Portugal; lcruz@deec.uc.pt; 3 Instituto de Telecomunicações, 3030-290 Coimbra, Portugal

**Keywords:** V-PCC compression, H.265/HEVC compression, packet losses, geometry and attribute point cloud measures, PCQM, image and video quality measures, SSIM

## Abstract

This article describes an empirical exploration on the effect of information loss affecting compressed representations of dynamic point clouds on the subjective quality of the reconstructed point clouds. The study involved compressing a set of test dynamic point clouds using the MPEG V-PCC (Video-based Point Cloud Compression) codec at 5 different levels of compression and applying simulated packet losses with three packet loss rates (0.5%, 1% and 2%) to the V-PCC sub-bitstreams prior to decoding and reconstructing the dynamic point clouds. The recovered dynamic point clouds qualities were then assessed by human observers in experiments conducted at two research laboratories in Croatia and Portugal, to collect MOS (Mean Opinion Score) values. These scores were subject to a set of statistical analyses to measure the degree of correlation of the data from the two laboratories, as well as the degree of correlation between the MOS values and a selection of objective quality measures, while taking into account compression level and packet loss rates. The subjective quality measures considered, all of the full-reference type, included point cloud specific measures, as well as others adapted from image and video quality measures. In the case of image-based quality measures, FSIM (Feature Similarity index), MSE (Mean Squared Error), and SSIM (Structural Similarity index) yielded the highest correlation with subjective scores in both laboratories, while PCQM (Point Cloud Quality Metric) showed the highest correlation among all point cloud-specific objective measures. The study showed that even 0.5% packet loss rates reduce the decoded point clouds subjective quality by more than 1 to 1.5 MOS scale units, pointing out the need to adequately protect the bitstreams against losses. The results also showed that the degradations in V-PCC occupancy and geometry sub-bitstreams have significantly higher (negative) impact on decoded point cloud subjective quality than degradations of the attribute sub-bitstream.

## 1. Introduction

Point clouds are a fundamental data structure in the field of 3D modeling and computer vision. They represent objects and scenes by a set of points in a 3D space, where each point position is conveyed by (3D) spatial coordinates, possibly complemented by color or other attributes. Point clouds are commonly generated using 3D scanning technologies, such as LiDAR (Light Detection and Ranging), structured light, or photogrammetry, and are becoming very important in a wide range of applications, such as in Virtual Reality (VR) and Augmented Reality (AR) systems [1], but mostly in remote sensing applications in autonomous vehicles, robotics, urban planning and others. Recently, the ISO/IEC/ITU MPEG group developed several point cloud coders, namely G-PCC (Geometry-based Point Cloud Compression), V-PCC (Video-based Point Cloud Compression), and a LiDAR-specific codec [2,3]. V-PCC works by projecting static or dynamic point clouds onto a set of 2D patches that are encoded using legacy video technologies, such as the H.265/HEVC and H.266/VVC video codecs [4,5]. Each 2D patch is represented by an occupancy map which indicates if a pixel is present in a 3D projected point, a geometry image that comprises the depth information (depth map), and a series of attribute images forming each 2D patch (R, G, B; luminance or other information). These three maps are encoded, creating three bitstreams which can be transmitted over a network to convey the point cloud information to a remote receiver/user. For more details about the operation of V-PCC, please check [6]. Although there are other point cloud codecs, for this work we chose V-PCC due to its top-ranked (lossy) encoding performance when encoding dense time-varying point clouds typical of VR and AR streaming applications. The alternative codec G-PCC, also from MPEG, is better suited for sparser point clouds and so was not chosen. Other point cloud encoders, such as Draco [7], were not considered as they are better suited to encoding meshes or do not support the encoding of dynamic point clouds.

The quality of the dynamic point cloud streaming experience can be affected by several factors, one of which are packet losses affecting the encoded and packetized bitstreams. Packet losses occur when packets of data are lost in transmission between the source and destination due to routing buffer overflow, wireless links error bursts, or single-bit errors causing packet corruption, resulting in the degradation of the quality of the dynamic point cloud rendition, or even complete interruptions in the streaming.

This article addresses two main research questions. The first one is how significant is the impact on decoded dynamic point cloud subjective quality of packet losses affecting bitstreams generated by encoding dynamic point clouds with the MPEG V-PCC codec. Towards this objective the study presents analyses of the effects of different packet loss rates applied to different combinations of the V-PCC sub-bitstreams (attribute, occupancy and geometry), combined with different compression rates.

The second research question concerns the performance of several point cloud objective quality measures when used to evaluate the quality of the dynamic point clouds decoded from the pristine and impaired packetized V-PCC bitstreams. This performance assessment was based on correlation analyses between subjective quality scores obtained through a quality study and the values predicted by the different objective quality measures.

This article is organized as follows. Section 2 reviews the most relevant works related to video quality packet losses effects with and emphasis on V-PCC coding. Section 3 describes the procedure followed to process the subjective scores and the different correlation measures used. Section 4 provides the details of the subjective dataset creation, including compression and stream corruption with different packet loss rates (PLRs). This section also describes the evaluation setups used in the two labs involved in the study, one at University of Coimbra (UC), Portugal, and the other at University North (UNIN), Croatia, and presents the inter-laboratory correlation analyses. Section 5 presents and reviews the quality measures used in for the point cloud evaluation, covering both image- and video-based and point cloud-specific quality measures. This section also presents and analyses the correlations between some of these quality measures values and the subjective MOS (Mean Opinion Score) scores obtained during the subjective evaluations. In the case of quality metrics specific for point clouds, separate results are given for the entire test set, as well as for the test set without attribute-only degradations. Section 6 discusses the results and finally Section 7 draws some conclusions from the data and discussions laid-out in prior sections and proposes some research activities to be pursued in the future.

The main contributions of this article are summarized as follows:

- Proposed a method to apply artificial packet losses to sub-streams representing dynamic point clouds compressed with V-PCC;
- Prepared and published a new dataset consisting of three dynamic point clouds compressed with V-PCC, subject to packet losses and annotated with MOS scores obtained at UC and UNIN laboratories. This dataset is publicly available at http://vpccdataset.dynalias.com, accessed on 23 March 2023;
- Performed a comprehensive comparative evaluation of several point cloud objective quality measures (based on different principles) on the newly created dataset.

## 2. Related Work

Several articles have discussed at length the problem of video-quality degradation assessment due to the packet losses, when using different video coding methods. In [8], the authors assessed the MPEG-4 video quality with packet losses limited to I frames. The authors evaluate the effects of single packet losses at various frequencies and at various loss distances (measured with Group of Pictures—GOP), concluding that having more than two single losses in a short period of time will result in unsatisfactory video quality.

The authors of [9] assessed H.264/AVC, H.265/HEVC, and VP9-Coded video bitstreams without and with packet losses. Subjective evaluations were performed and objective measures were calculated in order to understand the advantages and disadvantages of applying the various coding techniques for use in IPTV services.

Article [10] reports how packet losses affect the quality of video sequences compressed with different settings and resolutions. A dataset was created of 11,200 full HD and Ultra HD video sequences encoded with the H.264/AVC and H.265/HEVC encoders at five bit rates and a simulated packet loss rate (PLR) ranging from 0 to 1%. Regardless of the compression levels, subjective evaluation, and objective measures showed that the video quality declines as the packet loss rate increases. Moreover, as bit rate increases, the quality of sequences affected by PLR decreases.

In [11], the effectiveness of 16 existing image and video quality measures (PSNR, SSIM, VQM, etc.) in assessing error-concealed video quality was examined. In most cases, measuring the objective quality of the overall video is a better way to assess the error concealment performance, than using the visual quality of the error-concealed frame by itself.

Ref. [12] proposed XLR (Pixel Loss Rate), a new key quality indicator for video distribution applications. The suggested indicator offers comparable results with existing full reference measures, incorporates the effects of transmission errors in the received video and has a high correlation with subjective MOS scores.

In order to measure the video distortion induced by packet losses for IPTV services, a parametric planning model incorporating channel and video properties was proposed in [13]. Video sequences were compressed using H.264/AVC encoding with different packet loss rates. According to the experimental results, the proposed model outperformed three widely used parametric planning models for video quality.

Ref. [14] presents a comprehensive overview of G-PCC and V-PCC rate-distortion coding performance. Static point clouds were compressed using default encoding configurations, and subjective evaluation was performed using a developed web-based system. State-of-the-art objective measures were also calculated to assess their capability to predict subjective scores.

In ref. [15] the authors compared two different options to perform on-line subjective quality of static point clouds, in the first participants download the entire dataset, while the second one uses a web-based solution. Both options showed strong correlation. Experiments were performed in the context of the Call for Evidence on JPEG Pleno Point Cloud Coding, comparing the existing point cloud coding solutions (G-PCC and V-PCC), and a deep-learning based point cloud codec. Subjective evaluation results were also compared with prior evaluations performed in laboratory settings (with the same test content).

Ref. [16] provides an overview of the JPEG Pleno Point Cloud activities, related to the Final Call for Proposals on JPEG Pleno Point Cloud Coding, for the evaluation of different existing point cloud coding solutions, G-PCC and V-PCC. Furthermore, different objective measures’ sensitivity to the point cloud compression artifacts was discussed.

Using the MPEG V-PCC standard codec, article [17] presents a study on the effects of simulated packet losses on dynamic 3D point cloud streaming. The authors showcased the distortions that occur when several channels of the V-PCC bitstream are lost, with the loss of occupancy and geometry data having the greatest negative effects on the quality. These findings highlight the need for more effective error concealing methods and the authors also described their experimental findings when two naive error concealing methods for attributes and geometry data were applied in the point cloud domain. Several objective measures have been also calculated, to compare them with point clouds without packet losses (i.e., only compressed). In [18], the same authors created a dataset with seven dynamic 3D point cloud sequences with various features, to determine the advantages and disadvantages of the newly proposed error concealing algorithms.

## 3. Subjective Scores Processing and Common Measures for Comparison

Subjective quality evaluation of point clouds can be performed using protocols similar to those used in image and video quality assessment, e.g., ITU-R BT.500-14 [19]. Point clouds may be evaluated subjectively in a variety of ways, such as through active or passive presentation [20], different viewing methods (such as 2D, side-by-side 3D, auto-stereoscopic 3D, immersive video, etc.) [21] raw point clouds, or point clouds after surface reconstruction [22]. Surface reconstruction is sometimes used to ease point cloud observation and grading, but surface rendering may result in undesirable effects unrelated to compression or take too long to compute for point clouds that are more complicated or noisy. A simpler and faster method to create watertight surface rendering of the point cloud data is to vary the point size (and possibly point type) to achieve the desired surface closure. According to the expected screen resolution, the distance and other camera settings for the virtual camera can also be adjusted to provide a point cloud rendering that facilitates its observation on a 2D screen and grading by human evaluators.

It is common to measure the degree of agreement between two sets of grades using correlation measures, such as Pearson Correlation Coefficient (PCC), Spearman’s Rank Order Correlation Coefficient (SROCC), and Kendall’s Rank Order Correlation Coefficient (KROCC) [23]. The grades under comparison can be MOS scores from different laboratories or series of MOS scores and corresponding objective scores.

In order to more closely match two sets of grades while utilizing PCC (representing either MOS scores from different laboratories or objective measurements with subjective MOS scores), a non-linear regression function is typically used. Alternatively, linear regression can be used to compare two sets of grades albeit loosing the ability to model more complex relationships.

Equations (1)–(4) define three non-linear fitting functions ($C_{1}$, $C_{2}$, and $C_{3}$), and a linear (affine) fitting function ($C_{4}$) that will be used later.

(1) $$C_{1}\left(z\right)=b_{1}\left(\frac{1}{2}-\frac{1}{1+e^{b_{2}\left(z-b_{3}\right)}}\right)+b_{4}z+b_{5}$$

(2) $$C_{2}\left(z\right)=\frac{b_{1}-b_{2}}{1+e^{\left(z-b_{3}\right)/b_{4}}}$$

(3) $$C_{3}\left(z\right)=b_{1}z^{3}+b_{2}z^{2}+b_{3}z+b_{4}$$

(4) $$C_{4}\left(z\right)=b_{1}z+b_{2}$$

While processing MOS scores, outlier detection mechanisms are used as described in ITU-R BT.500-14 [19] to remove unreliable scores before statistical analysis. Firstly, according to Equation (5), kurtosis $\beta_{i}$ and standard deviation $s_{i}$ are calculated for all video sequences *i*∈{1,n}, where $x_{i,j}$ are grades from all observers *j*∈{1,m} for video sequence *i* and $\bar{x_{i}}$ is the mean value of all grades for video sequence *i*. Afterwards, a screening rejection algorithm is applied, as described in Equation (6).

(5) $$\begin{array}{c} \beta_{i}=\frac{\frac{1}{m}\sum_{j=1}^{m}{\left(x_{i,j}-\bar{x_{i}}\right)}^{4}}{{\left(\frac{1}{m}\sum_{j=1}^{m}{\left(x_{i,j}-\bar{x_{i}}\right)}^{2}\right)}^{2}}\\ s_{i}=\sqrt{\frac{1}{m-1}\sum_{j=1}^{m}{\left(x_{i,j}-\bar{x_{i}}\right)}^{2}} \end{array}$$

for every video sequence *i* ϵ{1,n}

for every observer *j*

if $2\leq \beta_{i}\leq 4$

if $x_{i,j}\geq \bar{x_{i}}+2s_{i}\;\mathrm{then}\;P_{i,j}=P_{i,j}+1$

if $2\leq \beta_{i}\leq 4$

if $x_{i,j}\geq \bar{x_{i}}+2s_{i}\;\mathrm{then}\;P_{i,j}=P_{i,j}+1$

if $x_{i,j}\leq \bar{x_{i}}-2s_{i}\;\mathrm{then}\;Q_{i,j}=Q_{i,j}+1$

else

if $x_{i,j}\geq \bar{x_{i}}+\sqrt{20}s_{i}\;\mathrm{then}\;P_{i,j}=P_{i,j}+1$

if $x_{i,j}\leq \bar{x_{i}}-\sqrt{20}s_{i}\;\mathrm{then}\;Q_{i,j}=Q_{i,j}+1$

for every observer *j*

(6) $$\begin{array}{cc} \; & \;\mathrm{if}\;\frac{P_{i,j}+Q_{i,j}}{n}>0.05\;\mathrm{and}\;\left|\frac{P_{i,j}-Q_{i,j}}{P_{i,j}+Q_{i,j}}\right|<0.3\;\mathrm{then}\;\mathrm{reject}\;\mathrm{observer}\;j \end{array}$$

Another goodness-of-fit measure is root mean squared error (RMSE), defined in ITU-T P.1401 [24] by Equation (7)

(7) $$\mathrm{RMSE}=\sqrt{\frac{1}{n-1}\sum_{i=1}^{n}{\left(x_{i}-fit\left(y_{i}\right)\right)}^{2}}$$

In Equation (7), *x* and *y* are two sets of grades, where *y* scores are fitted on *x* scores.

Outlier ratio (OR) is also used for comparison between two sets of grades, e.g., from two different laboratories, and is defined in ITU-T P.1401 [24] as a number of grades that satisfy Equation (8).

(8) $$|x_{i}-fit\left(y_{i}\right)|>={\mathrm{CI}}_{i}$$

In Equation (8), *x* and *y* are two sets of grades, where *y* scores are fitted on *x* scores, while CI is defined as Equation (9)

(9) $$\begin{array}{c} {\mathrm{CI}}_{i}=t\left(m-1\right)\frac{s_{i}}{\sqrt{m}} \end{array}$$

where *m* is the number of scores that have been collected for each video sequence, t(m−1) is the Student’s *t* inverse cumulative distribution function, for the 95% confidence interval, two tailed test, with m−1 degrees of freedom, and $s_{i}$ is the standard deviation for all the scores that have been collected for video sequence *i*.

Furthermore, outlier ratio (OR) and Root mean square error (RMSE) can be used to compare MOS scores and objective scores, using the same equations as for the subjective scores, (7) and (8), where $y_{i}$ represents objective score for video sequence *i* and $x_{i}$ represents MOS score for video sequence *i*.

## 4. Dataset Construction and Subjective Quality Evaluation

This section describes the dataset creation, as well as the subjective evaluation protocol. Inter-laboratory correlation results will be also presented.

### 4.1. Dataset Construction

**Figure 1:**
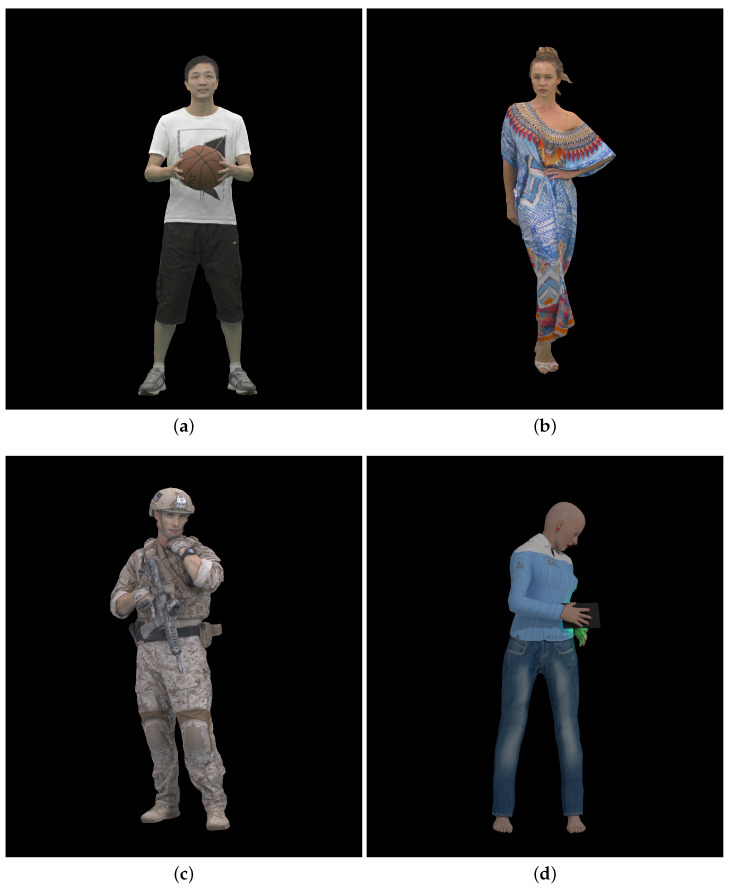
Reference point cloud visualization, first frame: (**a**) Basketballplayer; (**b**) Longdress; (**c**) Soldier; and (**d**) Queen.

We used three dynamic point clouds, Longdress and Soldier taken from the JPEG Pleno Point Cloud dataset [25,26] and Basketballplayer from [27] as the source contents to generate the test dataset. We used 300 point cloud frames for each of these three dynamic point clouds. In total, 250 point cloud frames of a fourth point cloud, Queen from Technicolor (https://www.technicolor.com/fr, accessed on 23 March 2023) (Creative Common Zero Universal 1.0 license (“CC0”)), were used in the subjective evaluation for user training at the start of each user session. Figure 1 shows 2D renditions of the first frame of each dynamic point cloud used in this work.

Dynamic point clouds were compressed using V-PCC compression software [3], with PccAppEncoder version 15.0. Default configuration files were used for each point cloud, creating five compression bit rates per point cloud. Group of frames (GOF) was set to the default value of 32 and the common test condition (CTC) parameters for the random access configuration were used. An example batch script for Basketballplayer point cloud, highest compression rate or smallest bit rate (r1) is presented in Listing 1.

**Listing 1.** V-PCC encoder batch script example.PccAppEncoder ^ --configurationFolder=cfg/ ^ --config=cfg/common/ctc-common.cfg ^ --config=cfg/condition/ctc-random-access.cfg ^ --config=cfg/sequence/basketball_player_vox11.cfg ^ --config=cfg/rate/ctc-r1.cfg ^ --frameCount=300 ^ --uncompressedDataFolder=basketball_player_vox11\ ^ --uncompressedDataPath=basketball_player_vox11_%%08i.ply ^ --reconstructedDataPath=reconstructed_1/basketballplayer_C01R01_rec_%%08d.ply ^ --compressedStreamPath=compressed_1/basketballplayer_C01R01.bin ^ --keepIntermediateFiles=1

Table 1 lists further details about the dynamic point clouds and the five compression rates used in the study.

**Figure 2:**
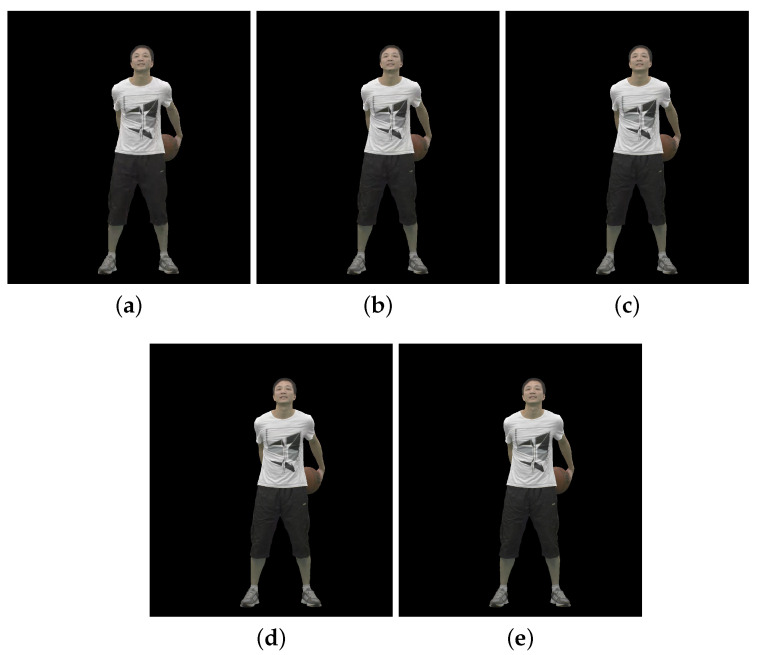
Basketballplayer point cloud without packet losses visualization, 30th frame: (**a**) compression rate 1; (**b**) compression rate 2; (**c**) compression rate 3; (**d**) compression rate 4; and (**e**) compression rate 5.

**Figure 3:**
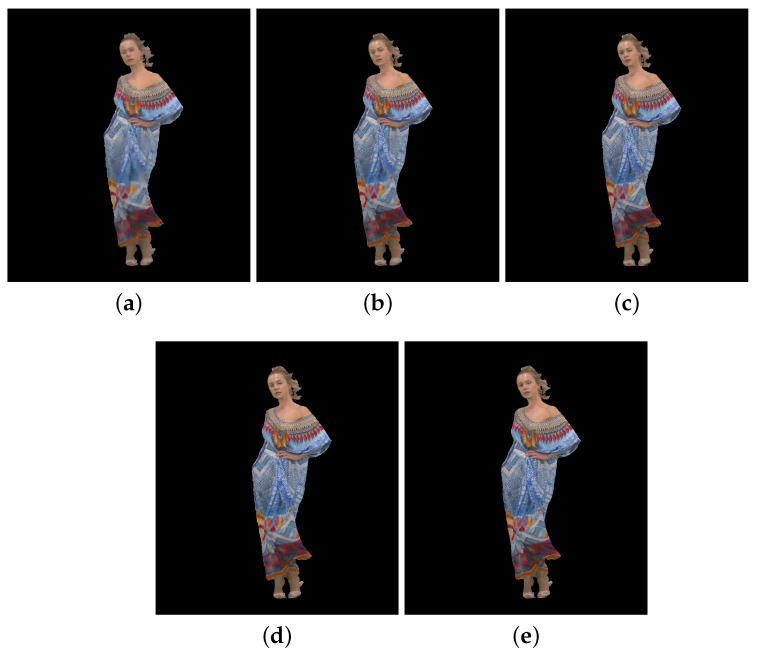
Longdress point cloud without packet losses visualization, 30th frame: (**a**) compression rate 1; (**b**) compression rate 2; (**c**) compression rate 3; (**d**) compression rate 4; and (**e**) compression rate 5.

**Figure 4:**
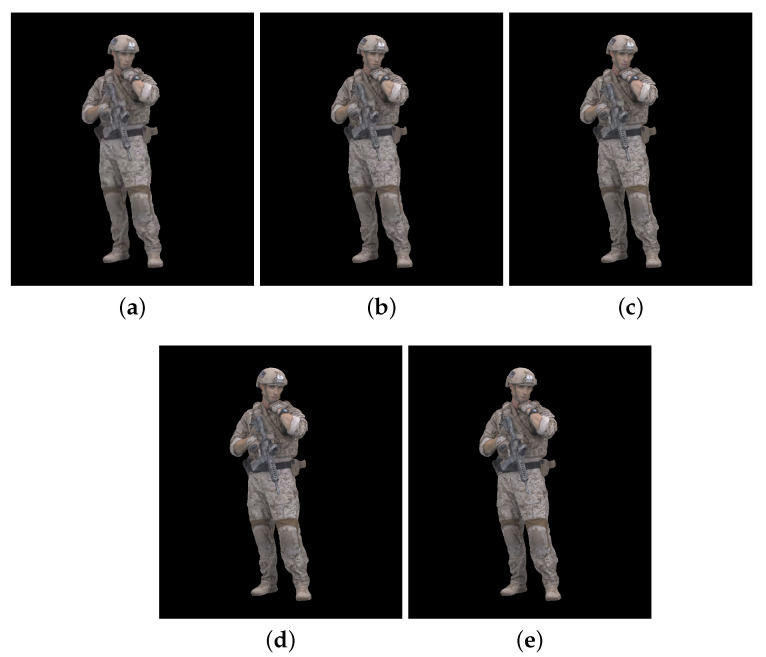
Soldier point cloud without packet losses visualization, 30th frame: (**a**) compression rate 1; (**b**) compression rate 2; (**c**) compression rate 3; (**d**) compression rate 4; and (**e**) compression rate 5.

Figure 2, Figure 3 and Figure 4 show the 30th frame from each point cloud compressed at the five rates indicated in Table 1. Even in this 2D representation the effects of the compression are clearly visible, especially if one compares the figures for rate 1 with the corresponding ones for rate 5.

The compressed dynamic point cloud streams are then processed by a H.265/HEVC bitstream transmission simulator based on Matlab [28]. The simulator offers three types of stream corruption:

- 0: corrupts all the packets according to the error pattern file;
- 1: corrupts all the coded packets but the ones containing intra coded slices;
- 2: corrupts only packets containing intra coded slices.

In this work, we used the first type of stream corruption which selects the packets to be marked as corrupt/lost according to the error pattern stored in a file. Prior to running the transmission simulator, Gilbert–Elliot error pattern files were prepared for the three target packet loss rates (PLRs) 0.5%, 1%, and 2% with different offsets. These patterns were created using a Matlab script implementing the Gilbert model [29]. Afterwards, the bitstreams were processed with the transmission simulator to produce the bitstreams that were then decoded using the V-PCC decoder (PccAppDecoder.exe). Details of the PLRs are given in Table 2. Because V-PCC encoder produces three bitstreams (for occupancy, geometry, and attribute), overall seven combinations of corrupted streams can be created. We used two combinations, attribute only (later called A) and occupancy + geometry + attribute (later called OGA). As tallied in Table 3, the dataset for subjective evaluation includes 3 reference point clouds (non-compressed) plus 3 (dynamic point clouds) × 5 (compression rates 1–5, 1 being smallest bitrate) × 2 (combination of corrupted bitstreams) × 3 (PLRs) = 90 packet-loss degraded dynamic point clouds plus 3 × 5 = 15 compressed dynamic point clouds without packet loss degradations, totalling 108 dynamic point clouds.

For the training session, we used Queen dynamic point cloud with 10 different combinations:

- 6: PLR 0.5% with compression rate 5, PLR 1% with compression rate 3 and PLR 2% with compression rate 1, with combinations attribute only and occupancy + geometry + attribute;
- 3: compression only degradations, with compression rates 1, 3, and 5;
- 1: reference point cloud.

To be able to automatically transmit corrupted bitstreams using H.265/HEVC transmitter simulator, V-PCC decoder source code from [30], file mpeg-pcc-tmc2/source/lib/PccLibDecoder/source/PCCVideoDecoder.cpp, is appended according to the Listing 2, before video decoding. Using one script, modified V-PCC decoder is run, writing and reading bitstream after 10 s, while the other script checks every half second for specific stream files for occupancy, geometry, and attribute bitstreams and corrupts them if needed using a simulator.

For example, as a result, in the first group of frames (GOF), which consists of 32 point clouds, corrupted point clouds are:

- For PLR 0.5%: 29–32;
- For PLR 1%: 29–32 (32nd point cloud being lost);
- For PLR 2%: 16–30.

**Listing 2.** V-PCC PCCVideoDecoder.cpp source code.**if** ( keepIntermediateFiles ) { bitstream.write( binFileName ); } // *pause for 10 s* std :: this_thread :: sleep_for( std :: chrono::milliseconds( 10,000 ) ); **if** ( keepIntermediateFiles ) { bitstream.read( binFileName ); } // *Decode video*

Video sequences are then created from reference and degraded dynamic point clouds using Technicolor point cloud renderer [31]. Specific camera path setup for each point cloud is presented in Table 4. Frame width and height were set to 1920 × 2160. Point clouds were centered in the bounding box, background color was set to black, FPS setting was set to 30, “display overlay” setting was disabled, “play the sequences” setting was enabled. Point size and type were default (point size: 1, point type: point).

After using the rendering software, raw uncompressed video files are created for each point cloud with resolution of 1920 × 2160 pixels and pixel format rgb48. FFmpeg [32] software is then used to convert and combine side-by-side reference and degraded video sequences, using libx264rgb video coder with crf 0 and pixel format rgb24 (both parameters needed for lossless compression of the rendered point cloud projections/views). We used 30 FPS, creating 10 s video sequences for Basketballplayer, Longdress, and Soldier point clouds and 8.33 s for the training Queen dynamic point cloud. Since the evaluation protocol used calls for an explicit identification of the source/non-degraded reference video, the word “REFERENCE” is overlaid on the video showing the reference point cloud, placed at the bottom of the screen. During the subjective evaluation, the reference point cloud position alternates between left and right (and vice versa for the degraded point cloud), so that half of the observers grade video sequences with reference shown on the left-half of the screen, and the other half on the right-half.

**Figure 5:**
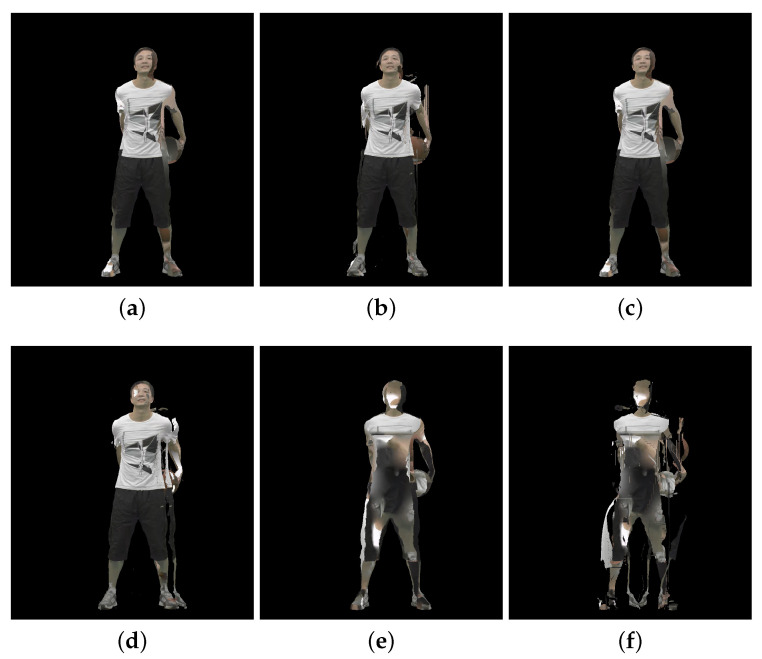
Basketballplayer point cloud with packet losses visualization, 30th frame, compression rate 3: (**a**) 0.5% packet losses: attribute only; (**b**) 0.5% packet losses: occupancy, geometry and attribute; (**c**) 1% packet losses: attribute only; (**d**) 1% packet losses: occupancy, geometry and attribute; (**e**) 2% packet losses: attribute only; and (**f**) 2% packet losses: occupancy, geometry and attribute.

**Figure 6:**
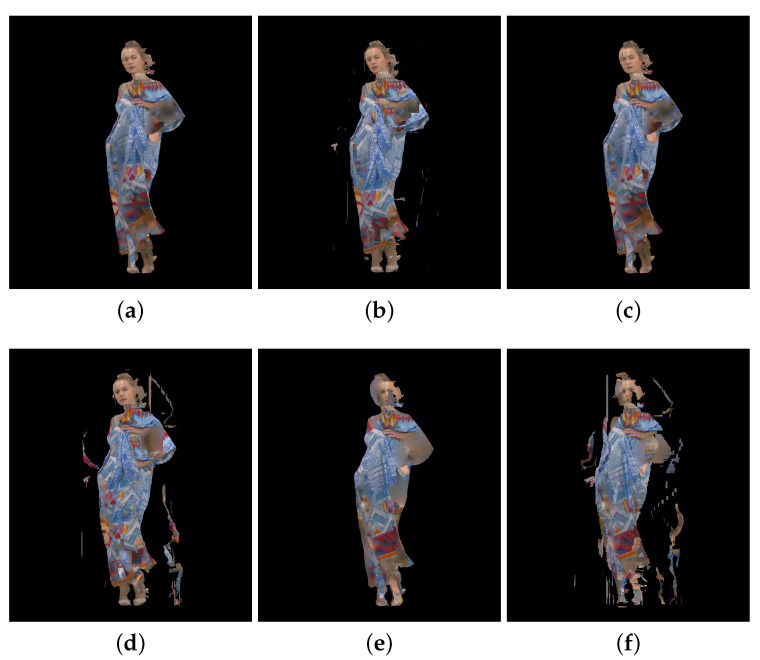
Longdress point cloud with packet losses visualization, 30th frame, compression rate 3: (**a**) 0.5% packet losses: attribute only; (**b**) 0.5% packet losses: occupancy, geometry and attribute; (**c**) 1% packet losses: attribute only; (**d**) 1% packet losses: occupancy, geometry and attribute; (**e**) 2% packet losses: attribute only; and (**f**) 2% packet losses: occupancy, geometry and attribute.

**Figure 7:**
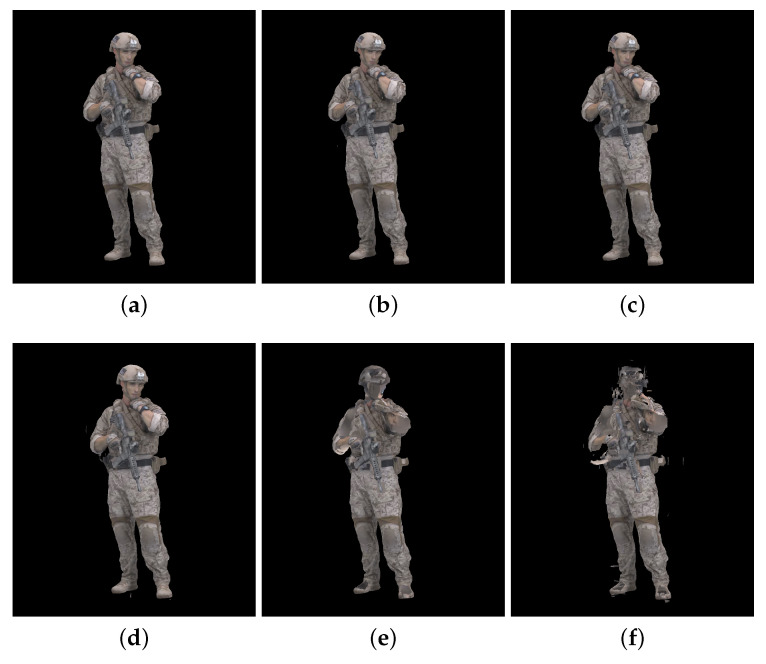
Soldier point cloud with packet losses visualization, 30th frame, compression rate 3: (**a**) 0.5% packet losses: attribute only; (**b**) 0.5% packet losses: occupancy, geometry and attribute; (**c**) 1% packet losses: attribute only; (**d**) 1% packet losses: occupancy, geometry and attribute; (**e**) 2% packet losses: attribute only; and (**f**) 2% packet losses: occupancy, geometry and attribute.

An example of 30th frame for Basketballplayer, Longdress, and Soldier point clouds are presented in Figure 5, Figure 6 and Figure 7, respectively. Each figure shows 3 packet loss rates with 2 types of corruption stream types (attribute only and occupancy + geometry + attribute) for each dynamic point cloud.

### 4.2. Subjective Experiments

In this subsection we will describe the protocol for subjective evaluation of the video sequences that were previously created from the point clouds. Two research laboratories were involved in this investigation, one at University of Coimbra (UC), Portugal, and the other at University North (UNIN), Croatia. We used a procedure similar to the one used in, e.g., [23,33] for static point clouds. Subjective evaluation was performed according to the protocols recommended by [19], using Double Stimulus Impairment Scale (DSIS) with a 5 level rating scale comparison between reference and degraded point cloud projected onto video frame. The scale levels measure the visibility and annoyance of the degradations relative to the original content according to: 1—very annoying, 2—annoying, 3—slightly annoying, 4—perceptible, but not annoying, 5—imperceptible. Hidden reference was also included for sanity check. Each observer graded 108 sequences in randomized order, with consecutive video sequences always displaying different point cloud models. A Matlab script was used to collect observers’ scores and to display video sequences using the MPV video player [34]. Training video sequences were shown at the beginning of the subjective evaluation, using 10 video sequences showing the Queen point cloud, to showcase the type and range of visible distortions.

**Figure 8:**
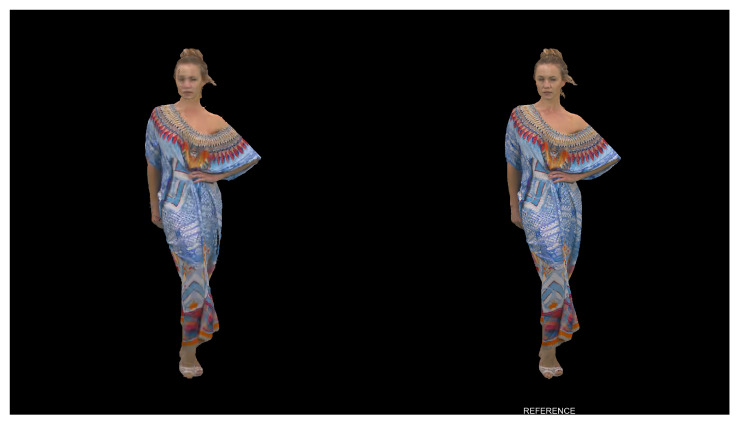
First frame from video sequence Longdress, compression rate 1.

Figure 8 shows an example of the screen as seen by an observer during the subjective evaluation. In this case a frame from video sequence Longdress, with reference frame on the right side, is shown. Each frame consists of 2 side-by-side point cloud projections with size 2 × 1920 × 2160 = 3840 × 2160 pixels.

The technical specifications of the equipment used as well as the demographic details of the observer pool are presented in Table 5. Since the screen used in the UC subjective evaluation had higher resolution than that of the videos, the MPV player command line was crafted to ensure it always displayed the video sequences at the original resolution (3840 × 2160 pixels), i.e., without rescaling.

**Figure 9:**
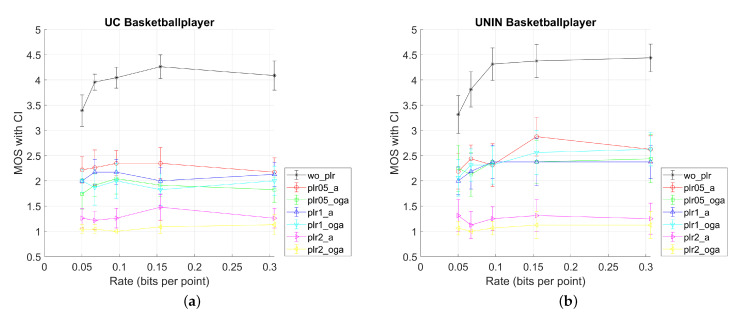

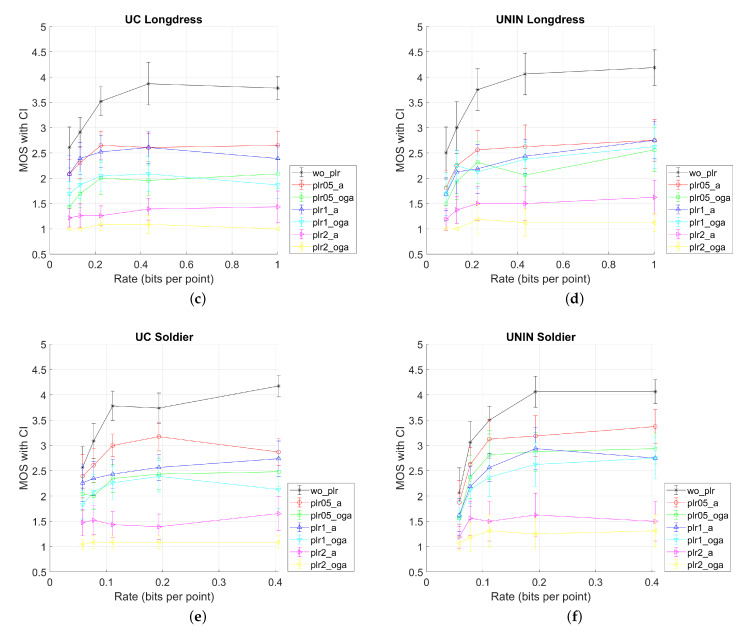
MOS results with CI values for three tested dynamic point clouds, from UC and UNIN laboratories: (**a**) UC Basketballplayer; (**b**) UNIN Basketballplayer; (**c**) UC Longdress; (**d**) UNIN Longdress; (**e**) UC Soldier; and (**f**) UNIN Soldier.

According to Equation (6) outlier rejection was carried out, but no outliers were found. Following that, MOS scores and CI were determined using Equation (9). Figure 9 show the results for UC and UNIN MOS scores. Additionally, outlier ratios are shown in Table 5.

When comparing MOS scores for UC and UNIN from Figure 9, it can be seen that results for UC have somewhat lower MOS scores for all tested video sequences, compared to the UNIN MOS scores. This difference may be due to the different screen size used in both laboratories.

Other results are as follows: best results are for only compressed sequences, with the lowest two compression rates (r4 and r5, Table 1) having similar MOS scores. For the PLR rate 2%, results are the worst for all compression rates, having constant low value independent of the compression rate. For this PLR rate, attribute (A) only corrupted stream has somewhat better MOS compared to the Occupancy + Geometry + Attribute (OGA) case of corrupted streams, but both being lower than 1.5. For the PLR rates of 0.5% and 1%, results are mostly similar, with PLR 0.5% and attribute (A) case having the highest MOS value. Furthermore, for the Basketball point cloud (with voxel depth 11 bits per dimension), results are similar for all compression rates, while for the Longdress and Soldier point clouds (with voxel depth 10 bits per dimension), MOS scores are somewhat higher for lower compression rates (r3, r4, and r5 from Table 1).

### 4.3. Inter-Laboratory Correlation Results

**Figure 10:**
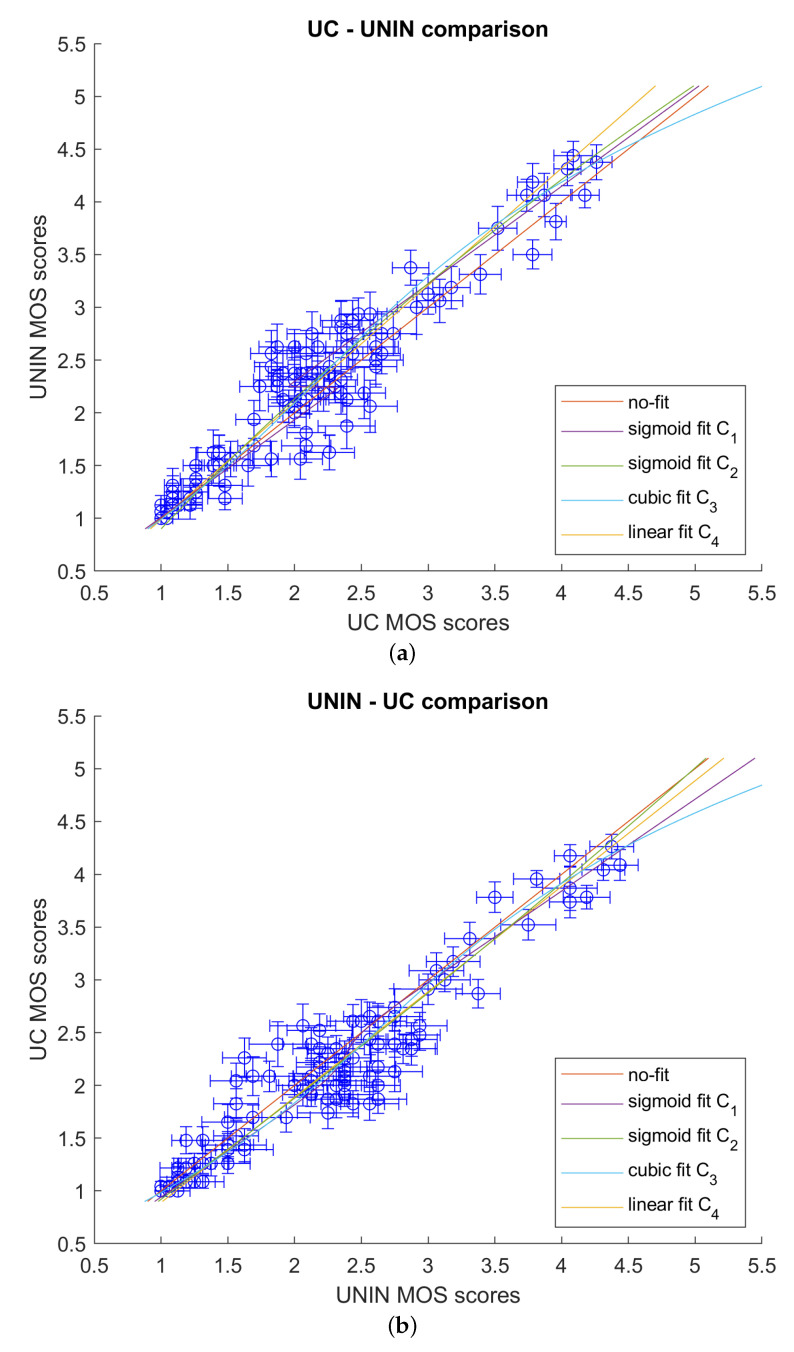
Inter-laboratory comparison: (**a**) UC-UNIN; and (**b**) UNIN-UC.

After that, correlations for the pairs UC-UNIN and UNIN-UC were computed using Equations (1)–(4) as fitting functions, and laboratories were compared. Table 6 and Table 7 give correlation results using PCC, SROCC, KROCC, RMSE (7), and OR (8). Figure 10 compares the two labs graphically. The results show a strong correlation between the two laboratories, demonstrating the accuracy of the subjective evaluation.

## 5. Objective Measures for Point Cloud Quality

In this section, we will firstly describe different image and video quality measures later used for correlation between objective and subjective MOS scores. Afterwards, different point cloud quality measures will be also explained. Finally, correlation results will be given for all tested objective measures.

### 5.1. Measures Based on Point Cloud Projections

If the point cloud is projected onto one or more 2D planes, general image objective quality metrics, such as Mean Squared Error (MSE), Peak Signal to Noise Ratio (PSNR), etc., can be used to determine the quality of the point cloud. In the case of a dynamic point cloud, video quality measures can also also used. For example, in [35], authors developed a rendering software for projecting a 3D point cloud onto a 2D plane. Afterwards, images of the point clouds were compared using different objective image quality measures, obtaining high correlation with subjective scores.

In this section, we will use several full reference image quality measures:

- MSE (Mean Squared Error), implementation from Matlab 64-bit 2020a;
- PSNR (Peak Signal to Noise Ratio), implementation from Matlab 64-bit 2020a;
- PSNRHVS (Peak Signal to Noise Ratio—Human Visual System) [36], implementation from [37];
- PSNRHVSM (Peak Signal to Noise Ratio—Human Visual System—Modified) [38], implementation from [37];
- SSIM (Structural Similarity index) [39], implementation from [40];
- MULTISSIM or MS-SSIM (Multi-scale Structural Similarity index) [41], implementation from [40];
- IWMSE (Information content Weighted Mean Squared Error) [42], implementation from [40];
- IWPSNR (Information content Weighted Peak Signal to Noise Ratio) [42], implementation from [40];
- IWSSIM (Information content Weighted Structural Similarity index) [42], implementation from [40];
- FSIM (Feature Similarity index) [43], implementation from [44];
- FSIMC (Feature Similarity index—Color) [43], implementation from [44].

Video quality measure that will be used is:

- VMAF (Video Multimethod Assessment Fusion) [45], implementation from FFmpeg [32] release 5.1.2, with the model “vmaf_v0.6.1.json”.

To create video sequences for the objective image and video quality measures, video sequences from the subjective experiments were used. Video sequences were divided into degraded and original video sequences using FFmpeg and saved as uncompressed “rawvideo” with pixel format “bgr24”, compatible with Matlab “VideoReader” function later used to import each video sequence. All image and video quality measures were calculated after image registration (degraded frame was registered onto the original), frame by frame, using Matlab with function “imregcorr”, which uses phase correlation. All measures except FSIMC use luminance component of the projected image (VMAF model “vmaf_v0.6.1.json” does not use chroma features [46]). The final image quality measure was calculated as a mean value from all 300 scores from each frame, overall 105 scores for later comparison with subjective MOS scores. In some cases, an empty frame was created from degraded video sequence (for 1% packet loss rate and “occupancy + geometry + attribute” combination of stream corruption), giving 9 NaN values for FSIM and FSIMC measures per video sequence. For those measures and those video sequences, mean value was calculated skipping all NaN values, i.e., using 291 scores.

When calculating average PSNR measure, results can be calculated in two different ways [47]:

- PSNR can be calculated from arithmetic mean of the MSE of the individual image in each video sequence: correlation results are similar to the arithmetic mean of MSE because it is actually scaled version of the arithmetic mean of MSE.
- PSNR can be calculated from average PSNR from all frames in each video sequence: this method was used to later report correlation between PSNR measure and subjective MOS scores. It is shown in [47] that this PSNR can be calculated from the geometric mean of the MSE of the individual image in each video sequence.

### 5.2. Geometry- and/or Attribute-Based Measures

Several point cloud objective quality measures have been recently proposed to quantify errors of the distorted point clouds, including V-PCC compression errors. Point cloud objective measures can be based on geometry and/or attribute information [23]. Some of the newly proposed point cloud objective measures that use both geometry and attribute information include full-reference PCQM (Point Cloud Quality Metric) [48], GraphSIM (Graph Similarity index) [49] and MS-GraphSIM (Multiscale Graph Similarity index) [50], reduced-reference RR-CAP (Reduced Reference Content-oriented Saliency Projection) [51], and no-reference SRG (Structure Guided Resampling) [52] objective measures.

To directly measure geometric and attribute distortions between two point clouds, we used several point cloud objective measures:

- Geometry: Point-to-point (p2p) [53],
  - L2 distance,
    ∗ RMS_p2p_, PSNR_RMS,p2p_;
  - Hausdorff distance,
    ∗ Haus_p2p_, PSNR_Haus,p2p_;
- Geometry: Point-to-plane (p2pl) [54],
  - L2 distance,
    ∗ RMS_p2pl_, PSNR_RMS,p2pl_;
  - Hausdorff distance,
    ∗ Haus_p2pl_, PSNR_Haus,p2pl_;
- Geometry: Density-to-density PSNR_D3_ [55],
- Geometry and attribute: PCQM [48] and MS-GraphSIM [50].

When utilizing point-to-point measures, the distance (error vector length) is determined between each point in the reference or degraded first point cloud and the point that is closest to it in the second point cloud (degraded or reference). Several distance definitions can be used; the two most common ones are Hausdorff distance and L2 distance. For L2 distance, a point-to-point distortion measure is calculated using the average (root) squared distances between point pairs. For Hausdorff distance, after calculating distance between all pairs of points, maximum squared distance is used as the calculated measure. The final measure, also known as a symmetric score, is typically defined as a measure with worse/higher score because point-to-point measure can be calculated in two different ways depending on the order of the point clouds (the first and second point cloud can be the reference and degraded point cloud or vice versa).

Point-to-plane measures, proposed in [54], use projected error vector (defined in point-to-point measures) onto unit normal vector in the first point cloud, calculating the dot product between them. Again, Hausdorff and L2 distance can be used. L2 Point-to-plane measure is calculated as the mean of the squared magnitudes of all projected error vectors, while Hausdorff distance point-to-plane measure uses maximum value of the squared magnitudes of all projected error vectors. The authors in [54] also proposed Peak Signal to Noise Ratio (PSNR), a novel metric that normalizes errors related to the largest diagonal distance of a bounding box of the point cloud.

PCQM is a new full-reference point cloud measure, proposed in [48]. Final PCQM score is calculated as a weighted combination of multiple features using data from both geometry-based and attribute-based point cloud features, where lower value represents smaller difference.

The density-to-density PSNR_D3_ measure described in [55] is based on the distortion of point cloud density distribution. It is designed to detect density distribution degradations, such as wrong occupancy estimation, which can occur in machine-learning point cloud coding solutions.

MS-GraphSIM measure, proposed in [50], divides local patches from reference and distorted point cloud into multiple scales and then fuses GraphSIM measure [49] at each scale into an overall MS-GraphSIM score. GraphSIM measure uses graph signal gradient to evaluate point cloud distortions by constructing graphs centered at geometric keypoints of the reference point cloud. Afterwards, three moments of color gradients are calculated for the same local graph, to obtain local significance similarity features. Finally, GraphSIM is calculated by averaging local similarity features across all color channels and all graphs.

For the geometry point-to-point and point-to-plane measures, we used software from [56], while for PCQM measure we used software from [57]. For MS-GraphSIM measure we used software from [58]. Final point cloud quality measure was calculated as a mean value from all 300 scores from each point cloud pair, overall 105 scores for later comparison with subjective MOS scores. For 1% packet loss rate and “occupancy + geometry + attribute” combination of stream corruption, an empty point cloud was created, giving 9 NaN values for geometry point-to-point and point-to-plane measures per point cloud sequence (“Cannot create a KDTree with an empty input cloud!” error in used application). For those measures and those point cloud sequences, mean value was calculated skipping all NaN values, i.e., using 291 scores.

### 5.3. Objective Image and Video Quality Measures and Correlation with MOS Scores

Correlation results between different image and video quality measures and MOS scores are given in Table 8 and Table 9 for UC and UNIN laboratories, respectively. For UC laboratory, best Pearson’s (PCC) correlation, using C_1_ (Equation (1)) and C_2_ (Equation (2)) functions for non-linear regression, is obtained with SSIM measure, while MSE is the second best. Best RMSE and OR with C_1_ regression function is also obtained with SSIM measure. Best Spearman’s (SROCC) correlation and Kendall’s (KROCC) correlation are obtained using MSE measure. For UNIN laboratory, best PCC correlation, using C_1_ and C_2_ functions for non-linear regression, is also obtained with SSIM measure, while FSIM measure has nearly the same correlation, being the second best. Best RMSE with C_1_ regression function is also obtained with SSIM measure. Best OR with C_1_ regression function is obtained with FSIM and FSIMC measures. Best SROCC and KROCC correlations are obtained using FSIM measure.

As described earlier, it should be noted that all results are calculated after image registration. Without this step, all correlation results, not presented here, are much lower.

**Figure 11:**
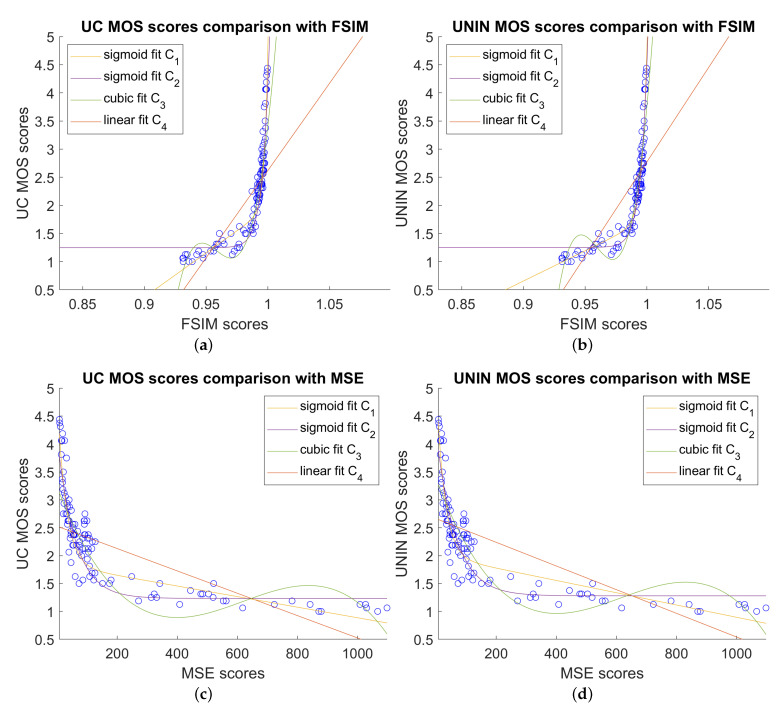

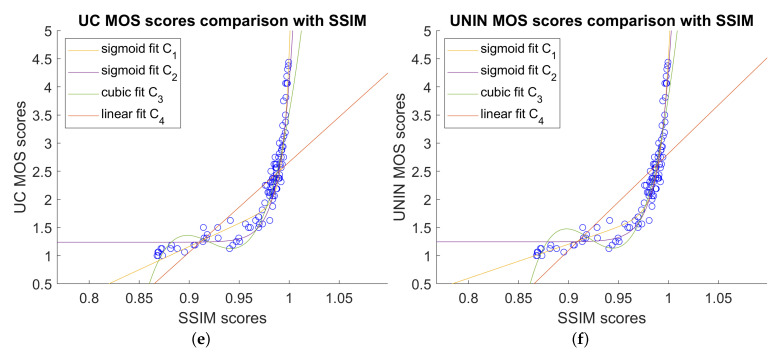
Comparison between best image quality measures and subjective MOS scores: (**a**) FSIM scores versus UC MOS scores; (**b**) FSIM scores versus UNIN MOS scores; (**c**) MSE scores versus UC MOS scores; (**d**) MSE scores versus UNIN MOS scores; (**e**) SSIM scores versus UC MOS scores; and (**f**) SSIM scores versus UNIN MOS scores.

Comparison between best objective measures FSIM, MSE, SSIM, and subjective MOS scores, for both UC and UNIN laboratories, are shown on Figure 11.

### 5.4. Objective Point Cloud Quality Measures and Correlation with MOS Scores

**Figure 12:**
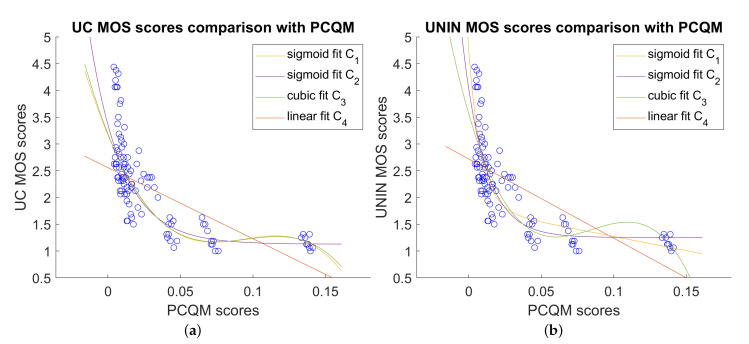
Comparison between best point cloud measures and subjective MOS scores, overall dataset: (**a**) PCQM scores versus UC MOS scores; and (**b**) PCQM scores versus UNIN MOS scores.

Correlation results between different point cloud quality measures and MOS scores for the overall dataset are given in Table 10 and Table 11 for UC and UNIN laboratories, respectively. In this case, PCQM measure gives the best results for all correlation measures (PCC, SROCC, KROCC) and RMSE. Only OR is the best for RMS_p2p_ point cloud measure for UC laboratory and PSNR_D3_ for UNIN laboratory. It should be noted that only PCQM and MS-GraphSIM detect both geometry and attribute errors, while other measures detect only geometry errors. Comparison between PCQM and subjective MOS scores, for both UC and UNIN laboratories, are shown on Figure 12.

Because most of the tested point cloud measures detect only geometry degradations, results are also given between point cloud quality measures and MOS scores for the dataset without only attribute degradations, which consists of 60 degraded point clouds. Those results are given in Table 12 and Table 13 for UC and UNIN laboratories, respectively. In this case Haus_p2p_ obtains the best correlation results in both laboratories for PCC with C_1_ (Equation (1)), C_2_ (Equation (2)) and C_3_ (Equation (3)) regression functions, as well as for RMSE comparison measure. Haus_p2pl_ is the second best measure for those measures in both laboratories.

RMS_p2p_ point cloud measure obtains the best correlation results in both laboratories for SROCC and KROCC correlation measures. Results for OR measure (with C_1_ regression function) are as follows, for UC laboratory, best and same results are for Haus_p2p_ and Haus_p2pl_ measures, while for UNIN laboratory, best results are for Haus_p2pl_ measure, with Haus_p2p_ being the second best.

**Figure 13:**
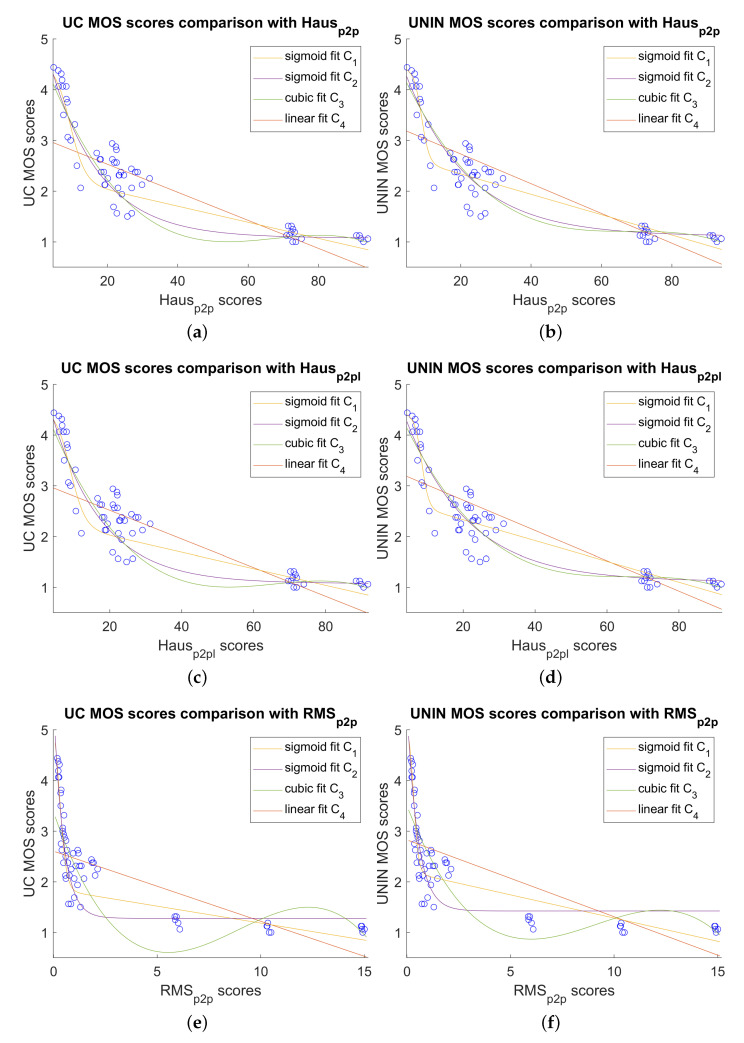
Comparison between best point cloud measures and subjective MOS scores without attribute only degradations: (**a**) Haus_p2p_ scores versus UC MOS scores; (**b**) Haus_p2p_ scores versus UNIN MOS scores; (**c**) Haus_p2pl_ scores versus UC MOS scores; (**d**) Haus_p2pl_ scores versus UNIN MOS scores; (**e**) RMS_p2p_ scores versus UC MOS scores; and (**f**) RMS_p2p_ scores versus UNIN MOS scores.

Comparison between best objective measure for the dataset without only attribute degradations, Haus_p2p_, Haus_p2pl_, RMS_p2p_ and subjective MOS scores, for both UC and UNIN laboratories, are shown on Figure 13.

## 6. Discussion

We compared the subjective quality MOS results from the UC and UNIN laboratories, using the correlation measures described earlier. Results show high inter-laboratory linear correlation (PCC), but a closer look at the ranges of the individual MOS scores shows that the UC MOS scores are somewhat lower than the UNIN MOS scores. As discussed earlier, this difference might be due to the different monitor types that were used in the subjective evaluation. It is possible that the evaluation protocol type used, DSIS (Double Stimulus Impairment Scale), could also have some impact on the final scores from the two laboratories. A similar protocol was used in earlier experiments with the static point clouds, with a camera rotating 360° around the object [15,16]. Since the dataset and MOS scores from both UC and UNIN laboratories have been recorded and made available to the public, hopefully in the future more results compiled at different laboratories will provide additional data that will allow a deeper understanding of the results presented here by confirming the results from one of the labs.

Concerning the correlations between the different image and video objective measures and the MOS scores, best results are obtained for SSIM, MSE, and FSIM/FSIMC measures, after image registration. This could be explained by the ability of those measures to detect abrupt quality variations between corrupted and non-corrupted frames, while also being able to detect changes due to the compression-only degradation. The results also confirm the importance of the image registration step, compensating the spatial offsets and scale changes caused by the point cloud geometry information. Without this step, correlation results are much lower, for all tested measures. It can be also noticed that for our dataset, average MSE correlates better with the subjective MOS scores, compared to the average PSNR, which has lower correlation. Similar conclusions can be found in [59], where the authors concluded that in the presence of channel errors, which produce distortions only in some frames, the average MSE (and from that value calculated PSNR) correlates better with the subjective tests than the average PSNR (computed from the PSNR of each frame). This study also concluded that for video compression only (source coding), results are similar for both methods of PSNR calculation. We cannot draw similar conclusion for out study because our dataset, includes only 15 sequences/point clouds with compression-only degradations (out of 105), preventing statistically significant conclusions.

On the other hand, some of the results of our work can be compared with those listed in [17], which also studies and discusses the effects of simulated packet losses on dynamic 3D point cloud streaming. In this article, objective metrics were calculated for several dynamic point clouds distorted with packet losses in different channels of the V-PCC bitstream. For Basketballplayer, Longdress, and Soldier point clouds Hausdorff distance and Geometry PSNR are better for attribute only degraded bitstream than for occupancy only and geometry only degraded bitstreams (although presented results are for the all-intra mode for V-PCC compression). The experiments in our work are similar as we also introduce losses to the occupancy, geometry and attribute streams and we also observed that packet losses in the former two (occupancy and geometry) have a much higher impact in quality than losses in attribute information.

Ref. [60] discusses the subjective and objective quality assessment of dynamic point clouds using V-PCC compression (packet losses were not used). In this work, two point clouds have been used (Matis and Rafa), in combination with four quality levels and four point counts. Although they tested different types of distortion (compression and point count reduction), correlation between objective and subjective scores have similar trends as in our dataset. Authors noticed that metrics computed using Hausdorff distance are performing better than others. In our dataset, without attribute-only packet losses, Haus_p2p_ and Haus_p2pl_ also perform the best in both laboratories for PCC, RMSE, and OR, and very good for other correlation measures. Still in the case of [60] color-based metric PSNR_YUV_ (point cloud based measure, i.e., based on the nearest points between them) with luminance to chrominance weight ratio of 6:1:1 showed the highest correlation with subjective scores. In our case projection-based luminance-only measures such as MSE, SSIM, and FSIM applied after registration showed high correlation results but with an inconsistent behavior on the entire dataset.

When the attribute-only degradation cases were excluded from the performance (correlation) calculations, Haus_p2p_, Haus_p2pl_, and RMS_p2p_ performed the best, possibly because of the same explanation as for the image quality measures. As it is the case with image quality measures MSE and PSNR, once again PSNR based point cloud measure shows lower correlation, compared to the RMS-based measure on which it is based. Furthermore, point-to-point measure RMS_p2p_ (which uses L2 distance) has higher correlation as a point-to-point measure, compared to the point-to-plane measure RMS_p2pl_. This might be explained by the instability of point normals estimation for some point clouds and point cloud sections, as it was also concluded in [60]. In the case of Haus measure (which uses Hausdorff distance), point-to-point Haus_p2p_ and point-to-plane Haus_p2pl_ have similar correlation results.

When comparing different point cloud-specific measures, PCQM measure performs the best for the overall dataset because it can grade both geometry and attribute errors, however with lower correlation results, when compared to the image quality measures. Recently the authors of [61] tested different compression algorithms (V-PCC,G-PCC and a deep learning GeoCNN codec) using nine different static point clouds. Compared to the subjective methodology used in this article, they also used DSIS methodology but with different camera trajectory around the point clouds under observation, using a helix-like rendering trajectory. Results for V-PCC compressed static point clouds show lower correlation with MOS scores using projection-based measures (including MSE, SSIM, and FSIM) and best correlation for PCQM measure. From this observation we can conclude that new point cloud-based objective measures need to be developed, because projection depends on the camera view and can fail to take into consideration occluded or partially occluded point cloud regions, resulting in an erroneous quality measure.

## 7. Conclusions

In this article, we describe a study aimed at understanding and measuring the effect on decoded point cloud quality of losses on the information of V-PCC bitstreams, as well as the ability of current image-based and point cloud-specific objective quality measures to correctly predict the quality of the degraded point clouds. The study involved preparing a new compressed dynamic point cloud dataset using V-PCC compression and bitstream corruptions taking the form of packet losses with different loss rates. Three dynamic point clouds (plus one for the training session) were compressed with five bit rates using V-PCC with H.265/HEVC video compression, creating three video streams per compressed video sequence, occupancy, geometry, and attribute streams. Attribute-only and all streams were subject to simulated transmission with loss using the transmission simulator tool in Matlab, with random access corruption modality and PLR rate of 0.5%, 1%, and 2%, creating six additional degraded video sequences per one compressed sequence. Overall, 105 degraded and 3 original dynamic point clouds were projected onto images and transformed to video sequences. Those video sequences were subjectively evaluated in two laboratories, showing strong inter-laboratory correlation results. Several state-of-the-art image, video, and point cloud objective measures were then computed and compared with subjective MOS scores. Among the image-based quality measures, FSIM, MSE, and SSIM showed the highest correlation among image and video quality measures. In the case of point cloud-specific measures, PCQM showed highest correlation among all point cloud measures for the overall dataset. If attribute-only degradations were not considered in the analysis, Haus_p2p_, Haus_p2pl_, and RMS_p2p_ performed the best.

In the future, different visualization technologies can be used to evaluate dynamic point clouds with compression degradation and packet losses, for example 3D side-by-side, 3D auto-stereoscopic, and virtual reality headset. Depth information should be generated in those cases. Active evaluation might be also performed, so that the observers can freely choose the camera position. New objective measures for geometry and color might be also developed, especially those calculated directly from point clouds, because in some cases the exact camera view might be unknown, e.g., due to user interaction with variation in point of view during the evaluation, so the usual image- or video-based quality measures based on projections might be unusable. No-reference point cloud measures might be also developed, for the cases where the reference point cloud is not available. Other point cloud compression codecs, for example Google’s Draco [7] and MPEG’s G-PCC, might be also used in the future experiments including compression and transmission errors.

## Figures and Tables

**Table 1:** Point cloud information and compression rates used with V-PCC encoding.

	Basketballplayer	Longdress	Soldier	Queen
Voxel depth (bits) per dimension	11	10	10	9
RGB attribute bits	24	24	24	24
Overall uncompressed bits per point (bpp)	57	54	54	51
Number of point clouds	300	300	300	250
Average number of points per point cloud	2,908,043	834,315	1,075,299	1,002,412
Compression rate r1 (bpp)	0.0502	0.0864	0.0581	0.0433
Compression rate r2 (bpp)	0.0671	0.1331	0.0774	0.0577
Compression rate r3 (bpp)	0.0957	0.2244	0.1114	0.0851
Compression rate r4 (bpp)	0.1545	0.4336	0.1934	0.1568
Compression rate r5 (bpp)	0.3063	1.0011	0.4058	0.3147

**Table 2:** Packet loss rate details. OGA represents occupancy + geometry + attribute, A represents attribute only stream corruption type.

	0.5%, OGA	0.5%, A	1%, OGA	1%, A	2%, OGA	2%, A
Corruption modality	all (0)	all (0)	all (0)	all (0)	all (0)	all (0)
Offset	20	20	16	17	1	1
True PLR	0.53	0.53	1.08	1.08	2	2
Burst length, BL	1.5	1.5	1.2	1.2	1.5	1.5
True burst length	2.7692	2.7692	2.0556	2.0556	2.6389	2.6389

**Table 3:** Statistics of point cloud dataset for subjective evaluation.

	Basketballplayer	Longdress	Soldier
Compression rates	5	5	5
Packet loss rates (PLRs)	3	3	3
corrupted bitstream types	2	2	2
only compressed	5	5	5
reference	1	1	1
overall	36	36	36

**Table 4:** Camera path setup for the point cloud rendering software.

	Index	Pos.x	Pos.y	Pos.z	View.x	View.y	View.z	Up.x	Up.y	Up.z
Basketballplayer	0	1024	1024	−5504	1024	1024	1024	0.0000	1.0000	0.0000
	300	1024	1024	−5504	1024	1024	1024	0.0000	1.0000	0.0000
Longdress	0	512	512	3840	512	512	512	0.0000	1.0000	0.0000
	300	512	512	3840	512	512	512	0.0000	1.0000	0.0000
Soldier	0	512	512	3840	512	512	512	0.0000	1.0000	0.0000
	300	512	512	3840	512	512	512	0.0000	1.0000	0.0000
Queen	0	512	512	−2816	512	512	512	1.0000	0.0000	0.0000
	250	512	512	−2816	512	512	512	1.0000	0.0000	0.0000

**Table 5:** Equipment information, observers statistics, and outliers.

	UC	UNIN
Monitor	Eizo CG319X 4K HDR	Sony KD-55x8505C
Screen Diagonal	31.1${}^{\prime \prime }$	55${}^{\prime \prime }$
Resolution	4096 × 2160 pixels	3840 × 2160 pixels
Viewing distance	0.9 m	1.5 m
Male Observers	17	10
Female Observers	6	6
Overall	23	16
Age range (years)	18–56	22–37
Average age (years)	26.7	26.6
Number of outliers	0	0

**Table 6:** Inter-laboratory correlation, UC-UNIN.

	C_1_	C_2_	C_3_	C_4_	No Fit
PCC	0.9610	0.9510	0.9520	0.9496	0.9496
SROCC	0.9152	0.9003	0.9003	0.9003	0.9003
KROCC	0.7848	0.7638	0.7638	0.7638	0.7638
RMSE	0.2256	0.2524	0.2499	0.2559	0.2923
OR	0.2762	0.3143	0.2762	0.3143	0.3714

**Table 7:** Inter-laboratory correlation, UNIN-UC.

	C_1_	C_2_	C_3_	C_4_	No Fit
PCC	0.9527	0.9498	0.9512	0.9496	0.9496
SROCC	0.9003	0.9003	0.9003	0.9003	0.9003
KROCC	0.7638	0.7638	0.7638	0.7638	0.7638
RMSE	0.2616	0.2693	0.2656	0.2698	0.2923
OR	0.2095	0.2190	0.2095	0.2095	0.2571

**Table 8:** PCC, SROCC, KROCC, RMSE, and OR between UC MOS scores and different image and video objective quality measures (best values are bolded).

	MSE	PSNR	PSNRHVS	PSNRHVSM	SSIM	MULTISSIM	IWMSE	IWPSNR	IWSSIM	FSIM	FSIMC	VMAF
PCC_C_1_	0.9443	0.7629	0.7249	0.7151	**0.9485**	0.8659	0.9274	0.7086	0.8265	0.9285	0.9268	0.5897
PCC_C_2_	0.9299	0.7648	0.7277	0.7199	**0.9318**	0.8371	0.9215	0.7090	0.7881	0.9105	0.9085	0.5893
PCC_C_3_	0.8693	0.7651	0.7262	0.7179	0.9050	0.8317	**0.9084**	0.7089	0.7876	0.8756	0.8751	0.5903
PCC_C_4_	0.6871	0.7629	0.7249	0.7151	0.7520	**0.7630**	0.7576	0.7083	0.7580	0.7277	0.7283	0.5883
SROCC	**0.9442**	0.7559	0.7232	0.7141	0.9181	0.8206	0.9093	0.7035	0.7760	0.8924	0.8906	0.5607
KROCC	**0.8133**	0.5852	0.5482	0.5382	0.7789	0.6541	0.7563	0.5223	0.5978	0.7463	0.7445	0.4012
RMSE_C_1_	0.2686	0.5277	0.5622	0.5705	**0.2585**	0.4083	0.5327	0.5759	0.4595	0.3031	0.3066	0.6591
OR_C_1_	**0.3905**	0.5905	0.6190	0.6381	**0.3905**	0.5238	0.6000	0.6762	0.5810	0.4286	0.4476	0.7238

**Table 9:** PCC, SROCC, KROCC, RMSE, and OR between UNIN MOS scores and different image and video objective quality measures (best values are bolded).

	MSE	PSNR	PSNRHVS	PSNRHVSM	SSIM	MULTISSIM	IWMSE	IWPSNR	IWSSIM	FSIM	FSIMC	VMAF
PCC_C_1_	0.9267	0.8225	0.7938	0.7865	**0.9678**	0.9176	0.9427	0.7854	0.8806	0.9670	0.9661	0.6986
PCC_C_2_	0.9114	0.8296	0.7980	0.7919	**0.9617**	0.9128	0.9399	0.7859	0.8771	0.9607	0.9598	0.6992
PCC_C_3_	0.8622	0.8307	0.7965	0.7895	**0.9438**	0.9085	0.9308	0.7855	0.8758	0.9292	0.9298	0.6993
PCC_C_4_	0.6904	0.8225	0.7938	0.7865	0.7669	0.8047	0.7752	0.7845	**0.8311**	0.7463	0.7471	0.6985
SROCC	0.9142	0.8481	0.8220	0.8155	0.9582	0.9154	0.9391	0.8070	0.8868	**0.9643**	0.9630	0.7055
KROCC	0.7761	0.6708	0.6385	0.6304	0.8455	0.7717	0.8054	0.6196	0.7212	**0.8548**	0.8518	0.5117
RMSE_C_1_	0.3233	0.4893	0.5233	0.5313	**0.2166**	0.3421	0.5436	0.5326	0.4076	0.2193	0.2223	0.6156
OR_C_1_	0.3048	0.5048	0.5048	0.5048	0.1429	0.2952	0.4667	0.4286	0.3905	**0.1238**	**0.1238**	0.5905

**Table 10:** PCC, SROCC, KROCC, RMSE, and OR between UC MOS scores and different point cloud objective quality measures, for the overall dataset (best values are bolded).

	RMS_p2p_	PSNR_RMS,p2p_	RMS_p2pl_	PSNR_RMS,p2pl_	Haus_p2p_	PSNR_Haus,p2p_	Haus_p2pl_	PSNR_Haus,p2pl_	PCQM	PSNR_D3_	MS-GraphSIM
PCC_C_1_	0.6069	0.5693	0.5889	0.5532	0.6092	0.5490	0.6093	0.5420	**0.7561**	0.5695	0.5561
PCC_C_2_	0.6064	0.5613	0.5876	0.5411	0.6017	0.5530	0.6005	0.5490	**0.7569**	0.5624	0.5564
PCC_C_3_	0.5996	0.5605	0.5837	0.5383	0.6019	0.5520	0.6008	0.5471	**0.7564**	0.5456	0.5629
PCC_C_4_	0.5231	0.5501	0.4736	0.5197	0.5843	0.5477	0.5839	0.5420	**0.6368**	0.5043	0.5561
SROCC	0.6005	0.5400	0.5990	0.5308	0.5807	0.5507	0.5797	0.5421	**0.7344**	0.4820	0.5916
KROCC	0.4695	0.3978	0.4594	0.3885	0.4479	0.4053	0.4460	0.3993	**0.5615**	0.3560	0.4256
RMSE_C_1_	0.6487	0.6709	0.6596	0.6799	0.6472	0.6822	0.6471	0.6859	**0.5341**	0.6708	0.6783
OR_C_1_	**0.4667**	0.5524	0.5810	0.6286	0.4952	0.5429	0.4952	0.5619	0.5810	0.5429	0.7048

**Table 11:** PCC, SROCC, KROCC, RMSE, and OR between UNIN MOS scores and different point cloud objective quality measures, for the overall dataset (best values are bolded).

	RMS_p2p_	PSNR_RMS,p2p_	RMS_p2pl_	PSNR_RMS,p2pl_	Haus_p2p_	PSNR_Haus,p2p_	Haus_p2pl_	PSNR_Haus,p2pl_	PCQM	PSNR_D3_	MS-GraphSIM
PCC_C_1_	0.5850	0.5882	0.5445	0.5760	0.5782	0.5603	0.5786	0.5606	**0.8260**	0.6292	0.6673
PCC_C_2_	0.5605	0.5874	0.5445	0.5755	0.5514	0.5595	0.5505	0.5557	**0.8207**	0.6290	0.6532
PCC_C_3_	0.5579	0.5882	0.5433	0.5759	0.5517	0.5592	0.5507	0.5550	**0.8116**	0.6277	0.6554
PCC_C_4_	0.5140	0.5818	0.4673	0.5643	0.5490	0.5567	0.5484	0.5519	**0.6727**	0.6005	0.6532
SROCC	0.5403	0.5884	0.5780	0.5883	0.5106	0.5468	0.5091	0.5385	**0.8514**	0.6174	0.7099
KROCC	0.4021	0.4406	0.4331	0.4372	0.3774	0.4047	0.3755	0.3991	**0.6727**	0.4784	0.5354
RMSE_C_1_	0.6978	0.6958	0.7216	0.7033	0.7020	0.7126	0.7017	0.7124	**0.4849**	0.6687	0.6408
OR_C_1_	0.5524	0.4667	0.5143	0.5143	0.5524	0.5143	0.5333	0.4952	0.4952	**0.4000**	0.5333

**Table 12:** PCC, SROCC, KROCC, RMSE, and OR between UC MOS scores and different point cloud objective quality measures, dataset without attribute only packet losses (best values are bolded).

	RMS_p2p_	PSNR_RMS,p2p_	RMS_p2pl_	PSNR_RMS,p2pl_	Haus_p2p_	PSNR_Haus,p2p_	Haus_p2pl_	PSNR_Haus,p2pl_	PCQM	PSNR_D3_	MS-GraphSIM
PCC_C_1_	0.9467	0.7345	0.8887	0.6939	**0.9772**	0.7382	0.9762	0.7819	0.7426	0.7240	0.5309
PCC_C_2_	0.9263	0.7292	0.8695	0.6863	**0.9718**	0.7611	0.9702	0.7526	0.7490	0.7113	0.5204
PCC_C_3_	0.8362	0.7315	0.7878	0.6851	**0.9650**	0.7604	0.9634	0.7521	0.7433	0.7113	0.5264
PCC_C_4_	0.6563	0.7172	0.5848	0.6635	**0.7955**	0.7382	0.7953	0.7328	0.5902	0.7112	0.5194
SROCC	**0.9385**	0.7634	0.9085	0.7377	0.9195	0.7845	0.9155	0.7724	0.7861	0.7324	0.5999
KROCC	**0.7926**	0.5780	0.7433	0.5493	0.7743	0.5963	0.7685	0.5872	0.6205	0.5654	0.4437
RMSE_C_1_	0.3122	0.6575	0.4442	0.6977	**0.2057**	0.6536	0.2102	0.6040	0.6489	0.6684	0.8211
OR_C_1_	0.4167	0.6333	0.7000	0.8167	**0.3000**	0.6833	**0.3000**	0.6167	0.8333	0.7833	0.7833

**Table 13:** PCC, SROCC, KROCC, RMSE, and OR between UNIN MOS scores and different point cloud objective quality measures, dataset without attribute only packet losses (best values are bolded).

	RMS_p2p_	PSNR_RMS,p2p_	RMS_p2pl_	PSNR_RMS,p2pl_	Haus_p2p_	PSNR_Haus,p2p_	Haus_p2pl_	PSNR_Haus,p2pl_	PCQM	PSNR_D3_	MS-GraphSIM
PCC_C_1_	0.9425	0.8075	0.9050	0.7629	**0.9517**	0.8164	0.9516	0.7906	0.8208	0.8330	0.6299
PCC_C_2_	0.8957	0.8052	0.8641	0.7623	**0.9356**	0.8156	0.9343	0.8088	0.8397	0.8295	0.6236
PCC_C_3_	0.8326	0.8066	0.7873	0.7626	**0.9310**	0.8151	0.9297	0.8085	0.8217	0.8300	0.6254
PCC_C_4_	0.6997	0.7900	0.6259	0.7423	0.8196	0.7951	0.8194	0.7906	0.6498	**0.8279**	0.6228
SROCC	**0.8976**	0.8190	0.8937	0.7909	0.8966	0.8210	0.8917	0.8100	0.8740	0.8556	0.6831
KROCC	**0.7525**	0.6507	0.7330	0.6198	0.7513	0.6461	0.7456	0.6370	0.7136	0.6999	0.5158
RMSE_C_1_	0.3332	0.5879	0.4239	0.6443	**0.3061**	0.5756	0.3063	0.6102	0.5693	0.5514	0.7741
OR_C_1_	0.4500	0.5167	0.5500	0.6333	0.2833	0.4833	**0.2667**	0.5500	0.6333	0.5333	0.7167

## Data Availability

The data presented in this study are openly available at http://vpccdataset.dynalias.com, accessed on 23 March 2023.
